# The “GEnomics of Musculo Skeletal Traits TranslatiOnal NEtwork”: Origins, Rationale, Organization, and Prospects

**DOI:** 10.3389/fendo.2021.709815

**Published:** 2021-08-16

**Authors:** Fjorda Koromani, Nerea Alonso, Ines Alves, Maria Luisa Brandi, Ines Foessl, Melissa M. Formosa, Milana Frenkel Morgenstern, David Karasik, Mikhail Kolev, Outi Makitie, Evangelia Ntzani, Barbara Obermayer Pietsch, Claes Ohlsson, Martina Rauner, Kent Soe, Ivan Soldatovic, Anna Teti, Amina Valjevac, Fernando Rivadeneira

**Affiliations:** ^1^Department of Internal Medicine, Erasmus University Medical Center, Rotterdam, Netherlands; ^2^Department of Epidemiology, Erasmus University Medical Center, Rotterdam, Netherlands; ^3^Department of Radiology and Nuclear Medicine, Erasmus University Medical Center, Rotterdam, Netherlands; ^4^Rheumatology and Bone Disease Unit, CGEM-IGMM, University of Edinburgh, Edinburgh, United Kingdom; ^5^ANDO Portugal, Évora, Portugal; ^6^Department of Surgery and Translational Medicine (M.L.B.), University of Florence, Florence, Italy; ^7^Department of Internal Medicine, Division of Endocrinology and Diabetology, Endocrinology Lab Platform, Medical University Graz, Graz, Austria; ^8^Department of Applied Biomedical Science, Faculty of Health Sciences, University of Malta, Msida, Malta; ^9^Azrieli Faculty of Medicine, Bar-Ilan University, Ramat Gan, Israel; ^10^Department of Mathematics, South-West University Neofit Rilski, Blagoevgrad, Bulgaria; ^11^Children’s Hospital, University of Helsinki and Helsinki University Hospital, Helsinki, Finland; ^12^Research Program for Clinical and Molecular Metabolism, Faculty of Medicine, University of Helsinki, Helsinki, Finland; ^13^Folkhälsan Research Center, Folkhälsan Institute of Genetics, Helsinki, Finland; ^14^Department of Hygiene and Epidemiology, Medical School, University of Ioannina, Ioannina, Greece; ^15^Department of Health Services, Policy and Practice, Center for Research Synthesis in Health, School of Public Health, Brown University, Providence, RI, United States; ^16^Centre for Bone and Arthritis Research, Institute of Medicine, Sahlgrenska Academy at University of Gothenburg, Gothenburg, Sweden; ^17^Department of Medicine III, Medical Faculty, Technische Universität Dresden, Dresden, Germany; ^18^Clinical Cell Biology, Department of Pathology, Odense University Hospital, Odense, Denmark; ^19^Clinical Cell Biology, Pathology Research Unit, Department of Clinical Research, University of Southern Denmark, Odense, Denmark; ^20^Department of Molecular Medicine, University of Southern Denmark, Odense, Denmark; ^21^Institute of Biostatistics, University of Belgrade, Belgrade, Serbia; ^22^Department of Biotechnological and Applied Clinical Sciences, L’Aquila, Italy; ^23^Department of Physiology, Medical Faculty University of Sarajevo, Sarajevo, Bosnia and Herzegovina

**Keywords:** osteoporosis, genomics, translational medical research, personalized medicine, collaborative learning

## Abstract

Musculoskeletal research has been enriched in the past ten years with a great wealth of new discoveries arising from genome wide association studies (GWAS). In addition to the novel factors identified by GWAS, the advent of whole-genome and whole-exome sequencing efforts in family based studies has also identified new genes and pathways. However, the function and the mechanisms by which such genes influence clinical traits remain largely unknown. There is imperative need to bring multidisciplinary expertise together that will allow translating these genomic discoveries into useful clinical applications with the potential of improving patient care. Therefore “GEnomics of MusculoSkeletal traits TranslatiOnal NEtwork” (GEMSTONE) aims to set the ground for the: 1) functional characterization of discovered genes and pathways; 2) understanding of the correspondence between molecular and clinical assessments; and 3) implementation of novel methodological approaches. This research network is funded by *The European Cooperation in Science and Technology* (COST). GEMSTONE includes six working groups (WG), each with specific objectives: WG1-*Study populations and expertise groups:* creating, maintaining and updating an inventory of experts and resources (studies and datasets) participating in the network, helping to assemble focus groups defined by phenotype, functional and methodological expertise. WG2-*Phenotyping:* describe ways to decompose the phenotypes of the different functional studies into meaningful components that will aid the interpretation of identified biological pathways. WG3 *Monogenic conditions - human KO models:* makes an inventory of genes underlying musculoskeletal monogenic conditions that aids the assignment of genes to GWAS signals and prioritizing GWAS genes as candidates responsible for monogenic presentations, through biological plausibility. WG4 *Functional investigations*: creating a roadmap of genes and pathways to be prioritized for functional assessment in cell and organism models of the musculoskeletal system. WG5 *Bioinformatics* seeks the integration of the knowledge derived from the distinct efforts, with particular emphasis on systems biology and artificial intelligence applications. Finally, WG6 *Translational outreach*: makes a synopsis of the knowledge derived from the distinct efforts, allowing to prioritize factors within biological pathways, use refined disease trait definitions and/or improve study design of future investigations in a potential therapeutic context (e.g. clinical trials) for musculoskeletal diseases.

## Origins

The Human Genome Project (HGP) catalysed a series of new discoveries and improvement of health care. Spending 13 years and about US$1 billion to complete the first sequence of a human genome, the HGP demonstrated extensive potential of research expertise networks (i.e., delivering results faster than anticipated and starting a new era for the field of genomic medicine). Technological innovation now allows to genotype on average ~1 million variants in hundred(s) of DNA samples per day for as little as 30 euros (€) per sample, and/or sequencing a human genome for less than €800 within 24 to 48 hours. For more than a decade now, researchers all around the world have been successful in identifying genomic leads with the hope of understanding the causes and developing cures for complex diseases, including osteoporosis and other musculoskeletal (MSK) diseases.

We are currently living an unprecedented period of genetic discoveries. It is now crucial that the hundreds (and soon thousands) of genetic findings undergo scrutiny through bioinformatics, in-silico and functional in-vivo follow-up. The ample amount of data collected from GWAS and (whole-exome) sequencing efforts have significantly enhanced our understanding of the genetic determinants of monogenic and complex traits (1, 2). With the advent of such high-throughput genotyping and sequencing technologies, coupled with policies of free public availability, researchers across the world now have access to considerable amount of *Big Data* needing to be mined with the potential of improving care of patients suffering MSK conditions. Yet, follow-up research tends to focus on a small fraction of the genes in the genome, with 20% of gene-related publications focusing on just 1% of all genes (3). The current challenge for biology is to disentangle the motivations behind “what gets studied next”. Are researchers putting money, time and effort into what is most important or urgent? Or rather, into more of the already identified genes just because they will be more likely to reliably win grants and plaudits? As such, the identification of an ample number of genetic factors underlying osteoporosis and related conditions highlight the large array of opportunities for genomic medicine applications (4, 5). Monogenic MSK conditions are also subject of intense investigation with high-throughput technologies, solving many, but also leaving a large fraction of unexplained conditions of still-to-be elucidated genetic origin. Knowledge derived from even more comprehensive GWAS based on very large sequenced reference panels (6–8), but also knowledge arising from monogenic disorders presenting with alteration of bone mass and fragility (9, 10) is increasingly providing novel insight into the key regulatory mechanisms governing skeletal physiology (11). Such amount of genetic discoveries pleas for the creation of a roadmap to organize the functional assessment of genes and biological pathways underlying MSK metabolism. The roadmap will ultimately highlight the opportunities to translate these discoveries into clinical applications (i.e., the development of new medications) and fulfil the promise of the HGP by bringing genomic medicine to the clinical setting.

**Figure 1:**
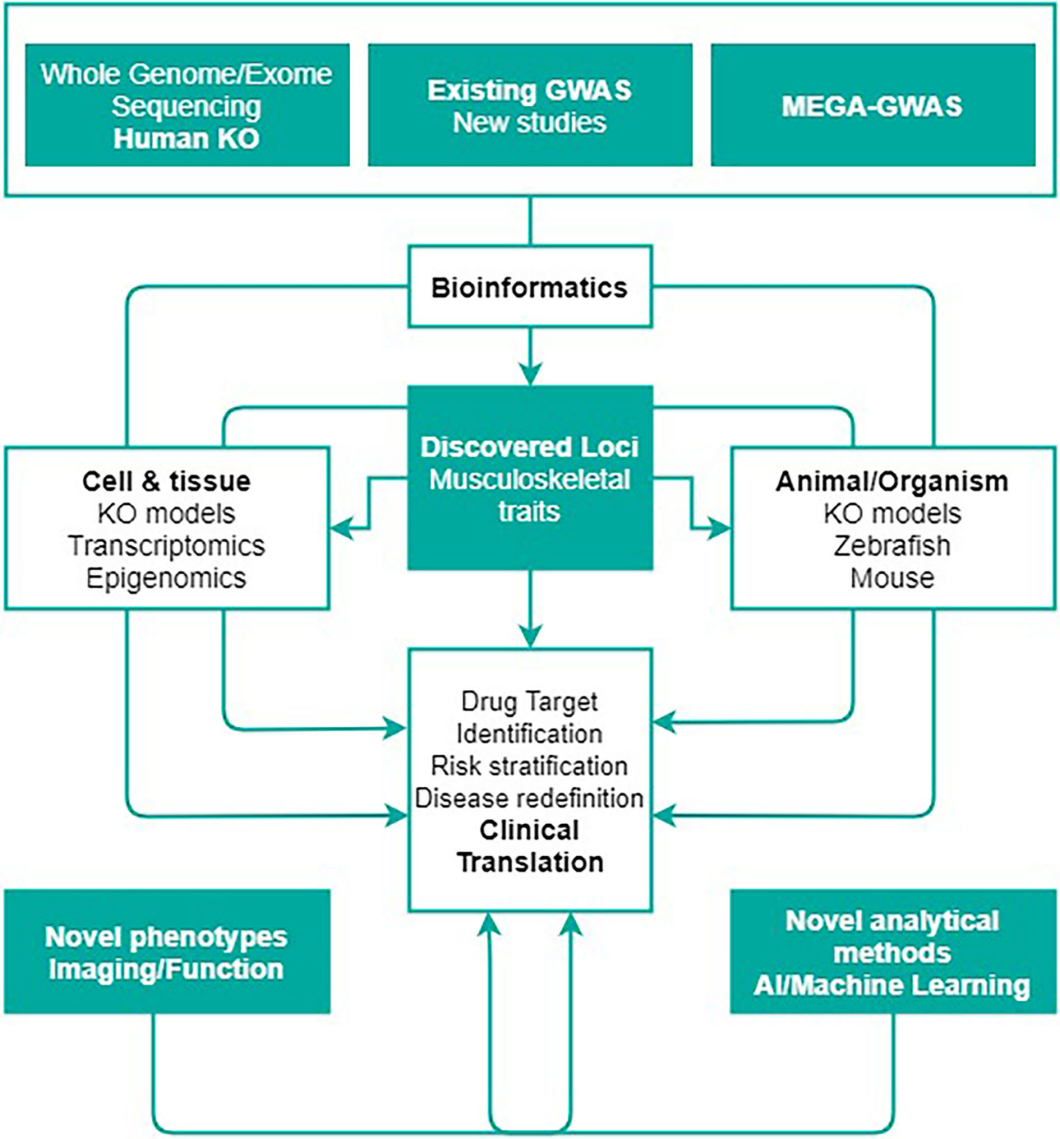
The abundance of genetic discoveries needs the creation of a roadmap seeking to organize the functional assessment of genes and biological pathways underlying musculoskeletal metabolism, ultimately highlighting the opportunities to translate these discoveries into clinical applications. This figure shows the rationale behind GEMSTONE scientific objective to translate current and future genetic findings in the clinic. Figure adapted with permission from Kiel et al. (12).

Collaboration between researchers working in the genomic, fundamental and clinical fields is crucial for successful translational efforts (**Figure 1**). Genomic research in the MSK field has brought together several scientists exploring most common MSK diseases [e.g., the Genetic Factors of Osteoporosis (GEFOS) (4, 6, 7) and the Genetics of Osteoarthritis (GO) consortia (8)]. Currently, there are numerous ongoing efforts in labs across the world to characterize genes and pathways that might be crucial to bone and cartilage metabolism. Frequently, these efforts do not follow leads provided by GWAS and/or embark on redundant assessments across labs, making inefficient use of resources. Further, there is an ongoing gap in publicly available resources, where cartilage and particularly bone cells and tissues are critically scarce and underrepresented, such as in the Encyclopaedia of DNA Elements (ENCODE) (13), the Genotype-Tissue Expression (GTEx) (14) and the RoadMap Epigenomics (15) projects.

**Figure 2:**
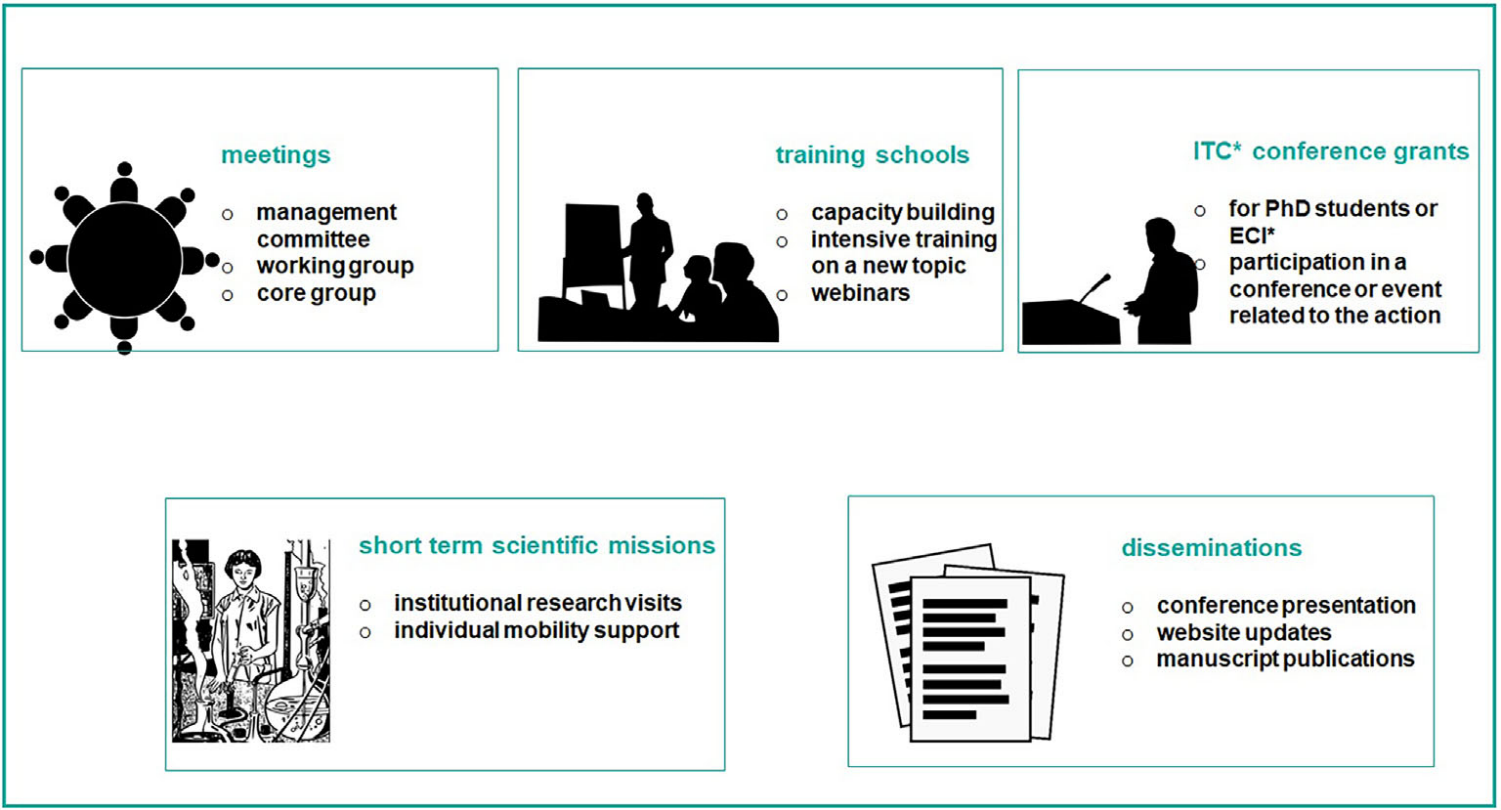
The figure shows networking activities funded by COST. They are exclusively dedicated to cover collaboration activities through specific networking tools, including workshops, conferences, working group meetings, training schools, short-term scientific missions, and dissemination and communication activities. *Acronym: ITC, inclusiveness target country; ECI, early career investigator.

The European Cooperation in Science and Technology (COST) is an organisation that funds the creation of research networks, called COST Actions (CA) (16). COST provides networking opportunities for researchers and innovators in order to strengthen Europe’s capacity to address scientific, technological and societal challenges. It aims at fostering interdisciplinary research for breakthrough science, promoting and spreading excellence and empowering and retaining young researchers and innovators. In addition, COST places emphasis on actively involving researchers from less research-intensive countries (Inclusiveness Target Countries, ITC). The funding provided by COST intends to complement national research funds, as it is exclusively dedicated to cover collaboration activities through specific networking tools, including workshops, conferences, working group meetings, training schools, short-term scientific missions, and dissemination and communication activities (**Figure 2**). The “GEnomics of MusculoSkeletal traits TranslatiOnal NEtwork” (GEMSTONE CA-18139) has brought together researchers from different disciplines working in the MSK field. GEMSTONE (17), aims at setting the ground for the: 1) functional characterization of discovered genes and pathways; 2) understanding of the correspondence between molecular and clinical phenotypic assessments; and 3) implementation of novel methodological approaches; within an integrative framework that will ultimately allow translating genomic discoveries into improved care for patients with MSK diseases.

## Rationale and Relevance

A compromised musculoskeletal system hampers mobility, a fundamental component of quality of life, health, and independence of individuals as they age. In the elderly, lack or limited mobility is a trigger for the development of numerous chronic conditions like osteoporosis, diabetes, hypertension and coronary heart disease. GEMSTONE aims to provide a basis for a better understanding of human musculoskeletal diseases with subsequent support of the development of new strategies for their prevention and therapy. GEMSTONE provides a platform where the expertise of researchers in the field of genetics and epidemiology is combined with that from researchers working on functional assessments, bioinformatics and clinical practice with the overall purpose of preventing musculoskeletal disease and improving patients’ treatment.

Current treatments for osteoporosis reduce non-vertebral fracture risk by only 25-50% (18–20) and there are concerns regarding side effects and long-term safety. Similarly, patients with osteoarthritis are asymptomatic in the early stages of disease, develop problems only after significant cartilage erosion has occurred, and no drugs are available to prevent or delay disease progression (21). While sarcopenia is now recognized as a medical condition (ICD10 code M62.84), there is still much understanding needed surrounding the definition, aetiology and potential treatments (22). Thus, there is an urgent need to advance understanding of musculoskeletal disorders, define new molecular pathways that facilitate development of new treatment options and also facilitate development of drug repositioning. The potential of incorporating genetic information in the search for suitable drug targets has recently been highlighted by a number of studies demonstrating how successful drug mechanisms are predicted by known genetic associations (i.e. the protein product modulated to elicit a clinical response). Such improved prediction is perceivable across the whole range of the drug development pipeline, from preclinical and clinical phases to launched drugs. One of these studies also showed that the highest degree of genetic support for drug-target indications was related to the musculoskeletal (bone mineral density), metabolic and blood categories (23). In this context, drug mechanisms with genetic support are shown to succeed twice as often as those without it (from phase I all the way to approval), and that is the case for osteoporosis drugs as illustrated in the same investigation (23).

The European Commission has supported well-conducted follow-up functional work focusing on genetic discoveries (24). Yet, these efforts are currently underway in a slow and competitive way. Such functional investigations are taking place within isolated efforts, which result in gaps of knowledge that slow down the field, in comparison to the proactive feedback and coupling that emerges from working in a network of multidisciplinary experts. GEMSTONE seeks collaboration with existing funded efforts, procuring to setup a network allowing the mining of resources extending from the cell (e.g., derived from human and mouse bone samples) to other organism (like mouse and zebrafish) models in a coordinated way. GEMSTONE is attempting this by bringing together diverse centres specialized in functional assessments (of the musculoskeletal system) using such cell and organism models (1, 2, 4, 6).

## Organization

GEMSTONE is organized across six focus working groups (WG) that include experts around the following research topics:

WG1 “*Study populations and expertise groups*” aims at creating, maintaining and updating an inventory of experts and resources such studies and datasets that participate in the network, helping to assemble focus groups defined by phenotype, functional and methodological expertise. The inventory collects information on the expertise of the distinct research groups active in musculoskeletal research across Europe and abroad, together with study membership. Such inventory allows to pinpoint focus groups defined by phenotype, functional and methodological expertise. Further, WG1 also seeks the identification of existing datasets that can constitute valuable resources where to mine information on genes and phenotypic assessments of the musculoskeletal system. The information collected by the inventory assembled by WG1 will be the foundation for allocating the appropriate emphasis and resources to follow-up studies and knowledge exchange, procuring to recruit and couple the different levels of expertise existing in the network. Ongoing synergy is established with the recently created Musculoskeletal Knowledge Portal (https://msk.hugeamp.org) (12, 25). This initiative, launched mainly by the International Federation of Musculoskeletal Research Societies (IFMRS) and The Broad Institute, provides an interface for researchers and clinicians to mine and follow-up the latest set of genomic discoveries under the light of functional investigations. GEMSTONE, specifically through the activities of WG1, is helping to put together an inventory of data resources that can enrich the portal.

WG2 “*Phenotyping*” aims to describe ways to decompose the phenotypes of different functional and clinical studies into meaningful components that might support the interpretation of identified biological pathways across the spectrum of models. A “think tank” has been assembled across different layers of expertise including clinical definitions of disease (i.e. fracture, sarcopenia, etc.), imaging applications and molecular definitions in humans, but also through the information derived from other cell and organism models on disease processes. The interaction of different members of the “think tank” enables the possibility of integrating various diagnostic approaches into one working definition of musculoskeletal disease. The activities of WG2 are crucial to establish the transportability of disease mechanisms and improving fidelity of animal models, both important steps to allocate resources to efforts that are most likely to have an impact on the understanding of disease mechanisms. Further, training on phenotyping techniques will be pursued across the expertise hubs of the network, seeking the setup of a phenotype map in the across cells/tissues, organisms, and human populations. On the long term, WG2 will continue working on tracing a phenotypic roadmap to dissect the heterogeneous components of disease into more homogeneous and molecularly-defined processes. This will ultimately allow a re-definition of musculoskeletal diseases (i.e., osteoporosis, osteoarthritis and sarcopenia) towards a better understanding of the underlying process and in this way having a better likelihood to tailor treatments in a translational context. This is one step away from the rule “one prescription fits all” and closer to palpable personalized medicine strategies. Precision medicine relies greatly on the successful integration between genomic data and clinical phenotypes (26).

WG3 “*Monogenic conditions- human KO models”* aims to make an inventory of all the genes underlying monogenic conditions presenting with a clear musculoskeletal phenotype. Studies of extreme phenotypes in humans have been instrumental in identifying molecular mechanisms underlying rare monogenic disorders as well as common and chronic diseases, including diabetes and obesity. Such studies have resulted in novel treatments that transform the lives of affected individuals (27–30). The biologic insight derived from genes identified through monogenic conditions is huge, as they constitute “human knock-outs” providing close to unequivocal evidence of involvement of a genetic cause of disease and open an array of translational opportunities. Application of an extreme phenotype approach to identify rare skeletal disorders in humans has led to discovery of SOST and LRP5 as critical regulators of WNT signalling in bone (31–33) and resulted in the development of new drugs to stimulate bone formation (34). The genes identified to underlie several bone disorders aggregating in families (typically identified through whole-exome and/or whole-genome sequencing) are starting to reveal a large overlap in the biologic pathways affecting monogenic and complex forms of musculoskeletal and other conditions (11, 35). From this perspective, factors identified through GWAS can be cross-validated through their implication in monogenic conditions, so-called human KO’s. Likewise, genes identified by GWAS to be involved in musculoskeletal outcomes, should primarily be considered for scrutiny as candidates to underlie such monogenic conditions.

WG4 “*Functional investigations*” aims to coordinate the functional evaluations across genes and pathways identified by genomic studies (both GWAS and sequencing), promote interaction and cross feedback between participating centres and specially limiting unnecessary redundancy and duplicate efforts across research teams embarking on functional follow-up. WG4 liaises with WG1 to make an inventory of the expertise across functional groups of the network with a trajectory on “wet lab” cell and organism models of the musculoskeletal system. Then, in cooperation with WG2, it will work on coupling and establishing correspondence between the phenotype characterizations at the human population and clinical case level and the different functional models arising from the cell and organism models. Finally, it aims to perform an assessment of the numerous genetic discoveries and procure classifying them into “functional units” within biological pathways but also considering the existing expertise across the groups. GEMSTONE seeks to seize the potential of setting up a synergistic collaboration between researchers capable of rapid-throughput skeletal phenotyping of a large set of identified genes knocked-out in cells, zebrafish and mouse models in relation to musculoskeletal phenotypes and place them in the context of investigations at the human population level. Such integration of resources will allow translating gene identification in model cells and organisms to human disease and vice versa. The results from the large-scale sequencing efforts and the GWAS require interpretation and functional follow-up, initially through the use of bioinformatics resources allowing high-throughput database mining of the discoveries; followed by the integration of functional studies on prioritized variants and candidate genes.

**Figure 3:**
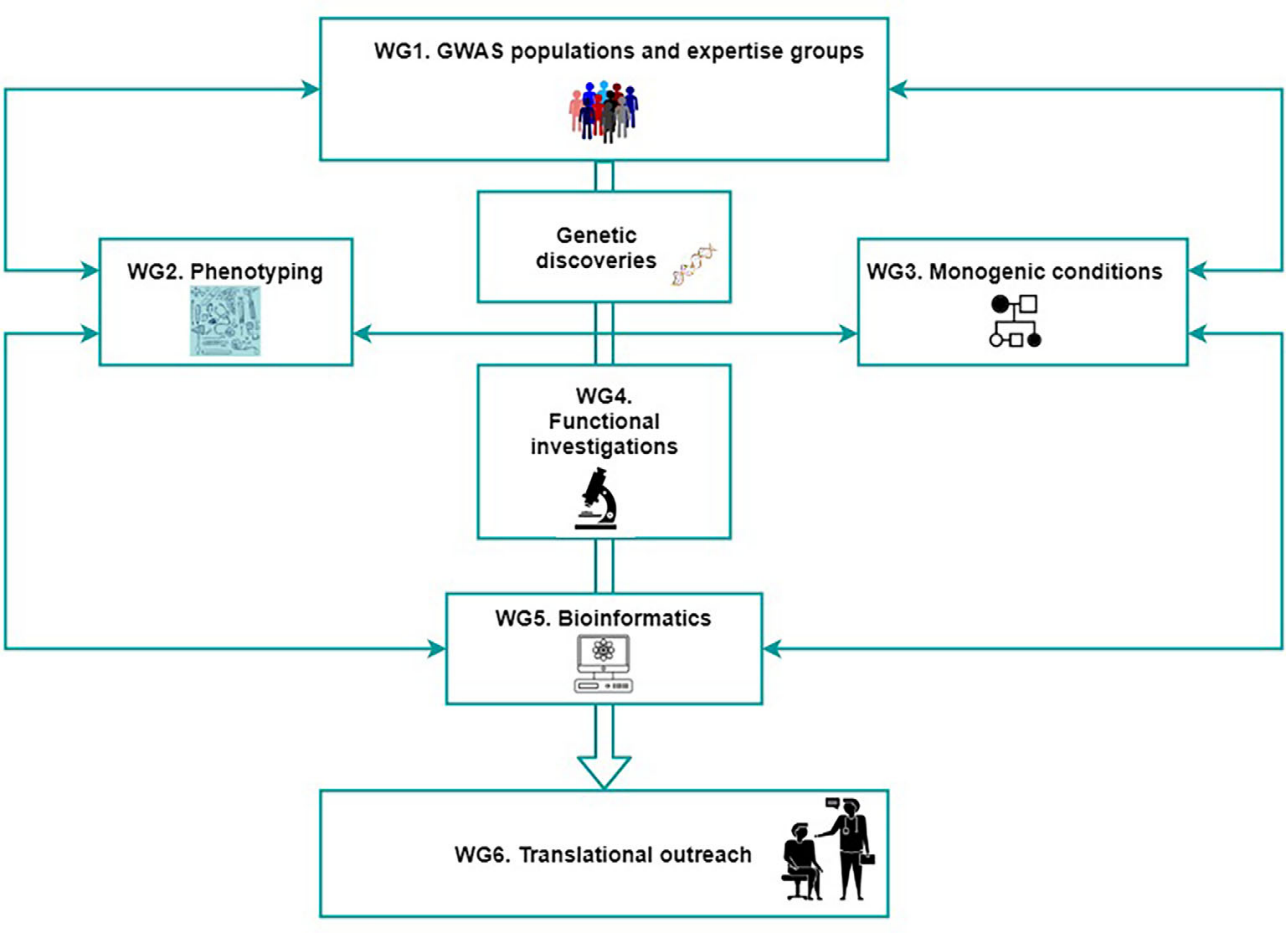
The organization of GEMSTONE across six working groups. Collaboration between researchers working in the genomic, fundamental and clinical fields is crucial for successful translational efforts. The figure shows the bridging of the different fields of science and disciplines of their members (genetic epidemiology, molecular biology, bioinformatics and clinical medicine), into one network that will allow advancing the field of musculoskeletal conditions by using such multidisciplinary approach. WG, working group.

WG5 “*Bioinformatics*” aims to make an inventory of all the genetic discoveries arising from the distinct efforts. This inventory will then be followed by the integration of databases and resources from the public domain, but also those assembled from the different functional working groups participating in the network. Such bio-informatic workup will procure facilitating the mining and characterization of the discoveries, extending from genetic variant to gene implication, to biologic pathway involvement, all up to phenotypic determination. WG5 will also procure bridging the different fields of science and disciplines of their members (genetic epidemiology, molecular biology, bioinformatics and clinical medicine), into one network that will allow advancing the field of musculoskeletal conditions by using such multidisciplinary approach (**Figures 1**, **3**). An expert panel will be created to distribute knowledge and skills from existing analytical approximations to the different working groups. Techniques employing standard statistical modelling will be put forward to participants of the network. The next step will be to train researchers on approaches of artificial intelligence (AI) techniques that have emerged as a tool to mine data for a deeper understanding and to assist clinical decision making. Such AI algorithms can use all the collected data from an individual to identify subtle and complex associations that are easily overlooked with traditional analytic approaches, such as multiple small deviations from the population mean in different parameters, which can act as a biomarker for early diagnosis of a disease or a marker of response to treatment. Human genetics can benefit immensely from AI launching a new era of *Big Data* possibilities.

WG6 “*Translational outreach*” aims to make a synopsis of the knowledge derived from the integration of activities and expertise provided by WG1, WG2, WG3, WG4, and WG5. Such synopsis will constitute a roadmap for the identification of drug targets by means of pinpointing molecules and factors within biological pathways that can be suitable for future investigations in a potential therapeutic context. Here GEMSTONE also ambitions to extend to other stakeholders, including funding agencies, involving the participation of several patient organizations and the pharmaceutical industry. In this way it can tackle the major hurdles hampering innovation in care and cure, which is needed to address the clinical needs of musculoskeletal disorders. The sequencing of the human genome was a game changer for researchers but even more for patients with genetic disorders, increasing their expectations of a solution in the horizon, in particular the ones with undiagnosed conditions. While GWAS of common diseases have delivered thousands of novel genetic findings, reaching the next level to identify the genes that are key for real outcome to patients, with the development of new treatments and repurposing current ones, has been a huge challenge. Yet, in all these steps of knowledge intake, patients are becoming more aware of the impact of their involvement in research. New patient experts have been engaging at diverse levels with basic science teams, academic institutes and medical centres, for early prioritization of research. Although still with some reluctance, the new paradigm of patient involvement in genetic studies, academic or industry led, is escalating towards a more efficient translational research. The patient is not always on the bedside, neither is the researcher alone by the bench. In this way, GEMSTONE is stepping towards aligning and connecting researchers conducting genetic studies on musculoskeletal traits, with patients, heading to a strong output.

## Prospects

**Figure 4:**
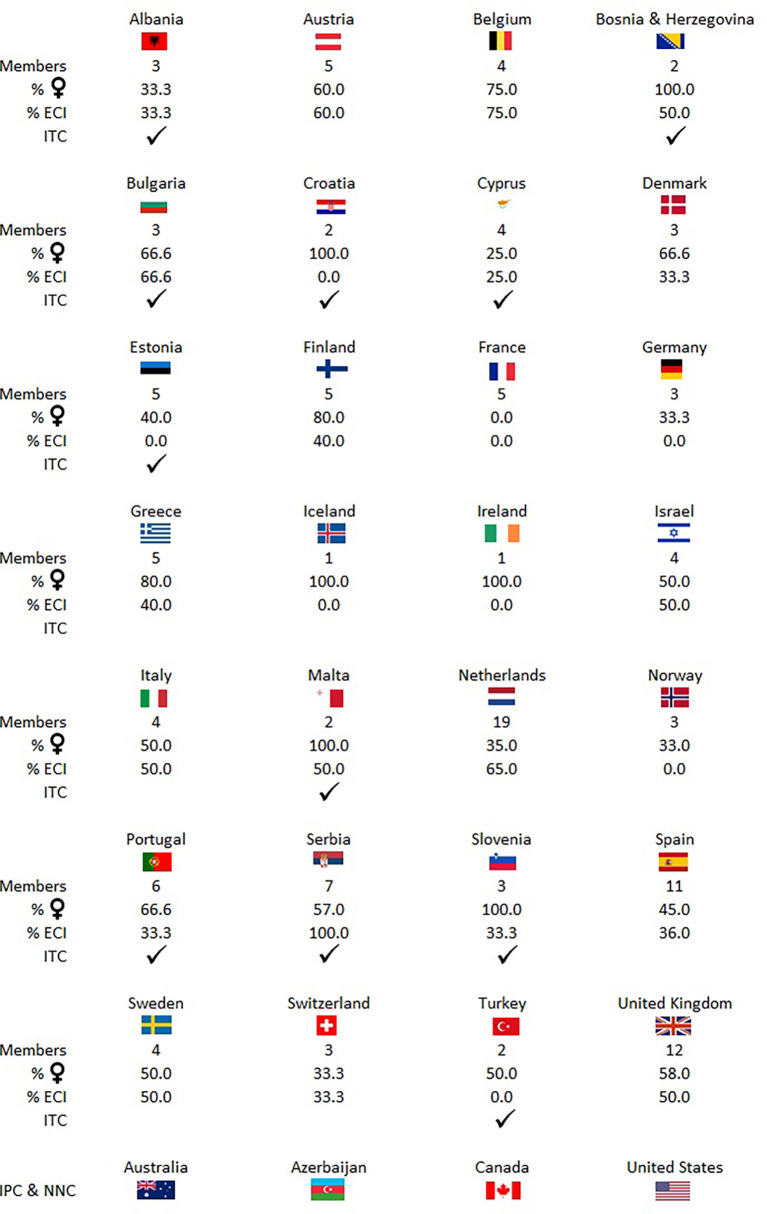
There are currently 140 researchers in the network coming from at least 32 countries inside and outside Europe. This figure shows the number of members, percentage of women, percentage of early career investigators (ECI) per country, and info on whether the given country is and inclusiveness target country (ITC). NNC, near neighbouring country; IPC, international partner country.

GEMSTONE is innovative in creating a unique multi-disciplinary network of researchers in the musculoskeletal field, combining together expertise on genetic epidemiology, molecular/systems biology, big data/bioinformatics and clinical medicine, which in a joint effort will bridge existing knowledge gaps. GEMSTONE is bringing an unprecedented number of experts in their specific fields into a network, working under a unified objective. There are currently 140 researchers in the network coming from at least 32 countries inside and outside Europe (**Figure 4**). The research expertise of the network’s members comprises medical sciences, biological sciences, genetics, bioengineering, epidemiology, mathematics, bioinformatics and chemistry among others. Several members of the GEMSTONE network of experts have embarked independently in sound scientific efforts albeit in many situations typically confined within a local (regional or even institutional) setting. To the disadvantage of the scientific community, a large fraction of these projects and derived resources will not be made publicly available. This can be the result of biobank regulations or simply due to lack of group membership to stimulate data sharing and newly generated knowledge. GEMSTONE seeks to provide a setup that will benefit those groups within Europe and abroad, from integrating such data and/or derived knowledge. As such, GEMSTONE brings together a unique scientific milieu envisioning the creation of a framework where several aspects of musculoskeletal disease are being set forward simultaneously in a coordinated way. These include trans-domain features, like patient involvement and cohort build-up, phenotype enhancement, outcome prioritization, and ultimately, the identification of prognostic biomarkers and the development of new drugs and assays. The establishment of a common phenotype database transcending the diagnosis of musculoskeletal conditions in humans and extending to the cell and organism level will allow exploring new genotype-phenotype relationships at a more comprehensive and yet unprecedented complexity. The partnership with the Musculoskeletal Knowledge Portal (12) described above, is an example of an interphase created to mine genomic discoveries. Such tool will allow bringing researchers and clinicians together around emerging knowledge on the biological mechanisms underlying musculoskeletal conditions; which in the future, can be translated to improved patient care (25).

To this end, in this current issue, GEMSTONE WG2 has shared with the readers a comprehensive overview provided by multidisciplinary experts in the musculoskeletal field entitled “*Translational approaches to musculoskeletal phenotyping across humans and animal models*” (36). The review describes the state-of-the-art technologies to investigate bone properties in humans and animals – including the methods’ strengths and weaknesses. They outline new research methodologies and future strategies to combine phenotypic with rapidly developing –omics data in order to advance musculoskeletal research and move towards “personalised medicine”. GEMSTONE WG3 also provides in this issue a review on monogenic forms of low and high bone mass disorders known to date, entitled “*A Roadmap to Gene Discoveries and Novel Therapies in Monogenic Low and High Bone Mass Disorders*” (37). As stated in the title, the review includes a roadmap to unravel the genetic determinants of monogenic rare bone mass disorders using proper phenotyping and genotyping methods, and describes different genetic validation approaches. GEMSTONE WG4 on the other hand has in this issue shared a consensus statement entitled “*Functional Validation for Skeletal Genetic Disease Using Cellular, Molecular and Animal-Modelling Techniques: A GEMSTONE Consortium Mission Statement*” (38). In the statement the WG4 members argue for the need to establish resource-sharing standards in order to promote data integration from various -omics technologies and functional dissection of human complex traits. They aim to create a roadmap for using functional genomics to interrogate signals from human genomic studies and to solve the pathogenesis of osteoporosis and other skeletal diseases at the molecular level. In the near future, WG5 and WG6 will provide their own reviews, summarizing the state-of-the-art in their fields of action, i.e., *Bioinformatics and Big Data analytics* (WG5) and *The Translational Outreach of Genomic Discoveries in the Musculoskeletal Field* (WG6).

GEMSTONE has been impactful primarily through the use of networking tools made available by COST (**Figure 2**). They help to advance the musculoskeletal field as a whole, through the direct actions and interactions of the network and the expansion of collaborative resources. Further, the dissemination of novel analytical approaches and access to technologies, countries, and/or institutions lacking them, helps to potentiate their new roles in more participative and active settings. Other aspects of translational outreach pursued by the GEMSTONE network include raising public awareness about musculoskeletal disorders. This way, GEMSTONE will help to shape the future musculoskeletal research agenda, encouraging translational opportunities derived from genomic discoveries, but also ensuring it includes the view of patients. The scientific progress achieved by the network will be disseminated in workshops and events that will include the general public, patients and patient organizations. More specifically, the scientific input will be shared with the general public through the GEMSTONE website and dedicated virtual platform with the help of the science communication coordinators. The scientific input will also be disseminated to the scientific community through participation of management committee members in annual meetings relevant to the musculoskeletal research field. GEMSTONE will communicate to society the importance of early detection and precise, tailored treatments for major musculoskeletal complex disorders. In due time, GEMSTONE will also be in the position of reaching out to patient organizations of rare monogenic musculoskeletal disorders, with an expanded list of variants in genes that should be considered for the assessment of still-unresolved cases.

GEMSTONE focuses also on capacity-building. By providing network opportunities to Early Career Investigators, GEMSTONE has enhanced the capacity building in the field of musculoskeletal disorders by connecting high-quality scientific communities throughout Europe and the world; and training young scientists from different fields and of different mind sets within the same framework. GEMSTONE is contributing to forming a generation of scholars and health professionals adequately trained and interlinked to rise up to the challenges of *Big Data* generation, handling, analysis and translation. Finally, GEMSTONE is expected to increase the impact of research on policy makers, regulatory bodies and national decision makers as well as on the private sector. From a health policy perspective, all the aforementioned impact aspects will ultimately lead to personalized, evidence-based, cost-effective treatments for patients suffering from musculoskeletal conditions.

## Author Contributions

All authors critically reviewed the article. FK and FR wrote the first draft of the manuscript. All authors contributed to the article and approved the submitted version.

## Conflict of Interest

The authors declare that the research was conducted in the absence of any commercial or financial relationships that could be construed as a potential conflict of interest.

## Publisher’s Note

All claims expressed in this article are solely those of the authors and do not necessarily represent those of their affiliated organizations, or those of the publisher, the editors and the reviewers. Any product that may be evaluated in this article, or claim that may be made by its manufacturer, is not guaranteed or endorsed by the publisher.
